# Interleukin-1/Toll-Like Receptor-Induced Nuclear Factor Kappa B Signaling Participates in Intima Hyperplasia after Carotid Artery Balloon Injury in Goto-Kakizaki Rats: A Potential Target Therapy Pathway

**DOI:** 10.1371/journal.pone.0103794

**Published:** 2014-08-01

**Authors:** Xiaotian Zhang, Yi Wang, Wenjing Hu, Dongye Li, Zhongmin Zhou, Defeng Pan, Wanling Wu, Tongda Xu

**Affiliations:** 1 Institute of Cardiovascular Disease Research, Xuzhou Medical College, Jiangsu Province, P. R. China; 2 Department of Internal Medicine, Aultman Hospital & Canton Medical Education Foundation, Northeast Ohio Medical University, Canton, Ohio, United States of America; 3 Cardiology of Affiliated Hospital of Xuzhou Medical College, Jiangsu Province, P. R. China; University of Iowa, United States of America

## Abstract

The value of restenosis after percutaneous coronary intervention (PCI) is recognized worldwide, especially for diabetic patients. Interleukin-1/Toll-like receptor (IL-1/TLR) signaling is involved in innate and adaptive immune responses, but whether and how the IL-1/TLR-induced nuclear factor kappa B (NFκB) pathway plays key roles in intimal formation is unclear. The underlying mechanism of intima hyperplasia was investigated with a model of carotid balloon injury in Goto-Kakizaki (GK) and Wistar rats and with lipopolysaccharide-stimulated macrophages. Elastic-van Gieson staining showed the medial area peakedon Day 3 post-injury and decreased by Day 7 post-injury in both GK and Wistar rats. The N/M at Day 7 in GK rats was significantly higher than in Wistar rats (p<0.001). The percent of 5-ethynyl-2′-deoxyuridine (EdU) staining-positive cells on Day 3 post-injury was greater than seen on Day 7 post-injury in GK and Wistar rats. The percent of EdU-positive cells on Days 3 and 7 post-injury in Wistar rats was less than that found in GK rats (p<0.01; p<0.05). NFκBp65 immunostaining had increased by Day 7 post-injury. Agilent Whole Genome Oligo Microarray verified that the IL-1/TLR-induced NFκB pathway was activated by carotid balloon injury. TLR4, IL-1 receptor associated kinase, inhibitors α of NFκB, human antigen R, c-Myc (Proto-Oncogene Proteins), EGF-like module-containing mucin-like hormone receptor-like 1 and Interleukin-6 were up-regulated or down-regulated according to immunochemistry, quantitative real-time PCR, Western blotting and Enzyme linked immunosorbent assay. Overall, we conclude that the IL-1/TLR-induced NFκB pathway participates in the intimal hyperplasia after carotid injury in GK and Wistar rats and that GK rats respond more intensely to the inflammation than Wistar rats.

## Introduction

Coronary artery disease (CAD) is a major cause of morbidity and mortality throughout the world [1], [2]. Diabetes mellitus (DM) amplifies the risk of cardiovascular events 4–6 fold. Cardiovascular events are responsible for 75% of all hospitalizations, and 80% of all deaths are of diabetic patients [3], especially non-insulin-dependent DM, which is wide spread among humans [4]. The use of percutaneous coronary intervention (PCI) for CAD patients has greatly improved their prognosis compared to the traditional therapy. However, restenosis after PCI has become a medical issue, occurring in 10–50% of procedures [5]. Intravascular ultrasound can also show neointimal proliferation results in higher rates of restenosis in diabetes mellitus after PCI. A more diffuse and accelerated form of atherosclerosis with smaller vessel size, long lesions, or greater plaque burden in diabetes mellitus, may result in an increased risk of neointimal hyperplasia and restenosis after stenting in these patients [6]. Increasing experimental and clinical evidence shows that inflammation drives restenosis [7], [8].

**Figure 1:**
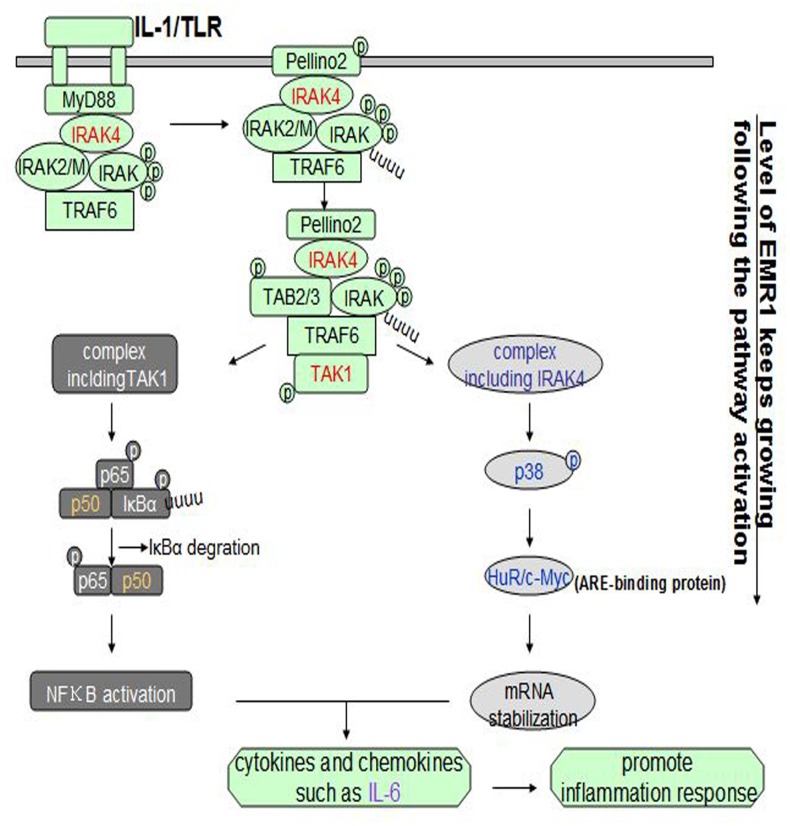
IL-1/TLR-induced NFκB signaling pathway. After the stimulation, IL-1R/TLR recruits adaptor molecule myeloid differentiation factor 88(MyD88) to their TIR domain, which further recruits and activates IRAK4. Then IRAK4, TRAF6 and IRAKs combine into a complex. After the coalition of Pellino2 and TAK1, the new complex is divided into at least two parts: complex including TAK1, activating NFκB through IκBα phosphorylation and degradation, and complex including IRAK4 which phosphorylates p38 and binds to the ARE-binding proteins like HuR and c-Myc. Two complexes both promote the release of cytokines and chemokines like IL-6 to further promote inflammation response. During the process EMR1 keeps growing.

That the Toll-like receptors (TLRs) are involved in innate and adaptive immune responses is well accepted. New functions for the interleukin-1/Toll-like receptor (IL-1/TLR)-mediated nuclear factor kappa B (NFκB) signaling pathway have been found [9] (Figure 1 [9]). However, whether and how this signaling pathway plays key roles in intimal formation after injury is unclear.

The Goto-Kakizaki (GK) rat is a well-characterized animal model for DM [4]. This electively inbred and nonobese strain was established by Goto and Kakizaki [10]–[12]. Response to the inflammation will be performed using this strain.

Based on this information, we developed a carotid injury model in Wistar and GK rats. The inflammatory response of bone marrow macrophages (BMM) fromthese rats was monitored to detect differences between the two strains and identify which signaling pathway is involved in the process. We hypothesized that IL-1/TLR-induced NFκB signaling is involved in the inflammation, with GK rats having enhanced neointimal proliferation compared with Wistar rats.

## Materials and Methods

### Animals

Fifty-seven GK male rats were used, of which 48 (300–330 g) were randomly separated into 3 groups: uninjured group (n = 18), Day 3 post-injury group (n = 12) and Day 7 post-injury group (n = 18). Three rats from each group were used for morphological and immunohistochemical analysis. Six rats from each group, with the exception Day 3 post-injury group GK rats, were used for expression analysis with an Agilent Whole Genome Oligo Microarray. Six rats were used for Western blotting experiments, and the remaining 3 rats were used for quantitative real-time PCR (qRT-PCR). Another 9 GK rats (150–180 g) were used for cell culture. Weight-matched non-diabetic Wistar rats, separated exactly like GK, were used as controls. All rats were fed a standard diet ad libitum. Room temperature was maintained at 23–25°C, with 50–60% humidity and 8 h period light daily. Animals and forage were purchased from SLAC Laboratory Animal Limited Company (Shanghai, China). The experimental procedures were carried out in accordance with the “Guide for the Care and Use of Laboratory Animals” (NIH Publication No. 85-23, National Academy Press, Washington, DC, revised 1996). The experiments were approved by the Animal Research Committee of Xuzhou Medical College (permit number, XMCACUC2010-08-114).

### Reagents

Both 5-ethynyl-2′-deoxyuridine staining (EdU) and the Cell-Light TM EdU Kit were purchased from Rui Bo Guangzhou Biotechnology Limited Company (China). The elastic-van Gieson staining (EVG) staining reagent was purchased from Baso Zhuhai Biotechnology CO. LTD. (China). The streptavidin-peroxidase (SP) immunehistochemical assay kit, 3, 3′-diaminobenzidine (DAB) kit and mouse anti-β-actin monoclonal antibody were manufactured by Zhongshan Goldenbridge Biotechnology CO. LTD (China). Antibodies against proliferating cell nuclear antigen (PCNA), TLR4 and IL-1 receptor associated kinase (IRAK4) came from Cell Signaling Technology (USA). Antibodies against Inhibitors α of NFκB (IκBα), NFκBp65, Human antigen R (HuR) and EGF-like module-containing mucin-like hormone receptor-like 1 (EMR1) were purchased from Santa Cruz Biotechnology (USA). Lipopolysaccharides (LPS) (Escherichia coli 055:B5) was obtained from Sigma-Aldrich. Fetal bovine serum (FBS), Dulbecco’s modified Eagle medium (DMEM) and 0.25% trypsin were purchased from Gibco. Interleukin-6 (IL-6) Enzyme linked immunosorbent assay (ELISA) kit was purchased from Western Tang. TRIzol reagent, the first-strand cDNA synthesis kit and SYBR Green Master Mix were purchased from Invitrogen.

### Methods invivo

#### Model preparation and samples collection

Rats were anesthetized by the intraperitoneal infusion of sodium pentobarbital (150 mg/kg). The left external carotid artery was exposed and a 2F Fogarty balloon catheter (Edwards Lifescience Corporation; USA) was introduced from the external carotid artery into the aortic arch after the head distal end of the external carotid artery was ligated. The balloon was distended pushed into the common carotid artery and withdrawn into the external carotid artery. The procedure was repeated 3 times before the external carotid artery was ligated. The left common carotid of rats was removed at Day 3 and 7 post-injury, while the left common carotid of uninjured rats was removed in both sets of rats. The specimens were stored in 4% paraformaldehyde prior to paraffin embedding or were frozen at −86°C.

#### Blood glucose and lipid levels

Blood samples acquired from each group of Wistar and GK rats before removal of the arteries were analyzed for serum glucose (GLU) levels, total cholesterol (CHOL) and triglycerides (TG) by the enzymatic method.

#### HE and EVG staining

After the specimens had been incubated in 4% paraformaldehyde for 24 to 48 h, they were embedded in paraffin. Paraffin sections (3–5 µm thick) were dewaxed, stained, examined microscopically and photographed. N/M was calculated with Image-Pro plus Version 6.0 for Windows (Media Cybernetics, Inc; USA).

#### EdU staining

Previously we had injected 100 mg/kg EdU (dissolved in 0.9% sodium chloride) intraperitoneally into rats [13]. Here. EdU 100 mg/kg was injected at 18, 12 and 2 h before removing carotid arteries. EdU-positive cells were examined by fluorescence microscopy (Olympus BX51 microscope; Japan). Images of cells dyed with Apollo 567 were captured with a ‘red’ filter, while Hoechst 33342-stained cell images were captured with a ‘blue’ filter. The percentage of EdU-positive cells was calculated using Image-Pro plus Version 6.0 for Windows (Media Cybernetics, Inc; USA).

#### Immunohistochemistry for PCNA and NFκBp65

Mouse monoclonal anti-PCNA and anti-NFκBp65 antibodies were the initial antibodies used and SP immunohistochemical assay kit was used during the entire staining procedure.

#### Agilent Whole Genome Oligo Microarray

Total RNA from each sample was quantified using a NanoDrop ND-1000 apparatus, and RNA integrity was assessed by standard denaturing agarose gel electrophoresis. RNA was amplified and transcribed into fluorescent cRNA using the Quick Amp Labeling protocol (version 5.7, Agilent Technologies). Labeled cRNA were hybridized onto the Whole Rat Genome Oligo Microarray (4×44 K, Agilent Technologies), and Agilent Feature Extraction software (version 10.7.3.1) was used to analyze acquired array images. Quantile normalization and subsequent data processing used GeneSpring GX v11.5.1 software package (Agilent Technologies). The roles of differentially regulated genes were identified by Pathway and GO Analysis. The distinguishable gene expression profiles among samples were examined by Hierarchical clustering.

(Microarray data are available at http://www.ncbi.nlm.nih.gov/geo/query/acc.cgi?acc=GSE48279).

#### qRT-PCR

Total RNA from each sample was extracted with the TRIzol Reagent and 2 µg RNA was used for a reverse transcription reaction using MMLV reverse transcriptase (Epicentre; Madison, WI). qRT-PCR was done with an ABI PRISM7900 system (Applied Biosystems, California, US). TLR4, IRAK4, HuR, IκBα, c-Myc and EMR1 expression was measured using 2×PCR master mix (Superarray), the results being normalized with the rat housekeeping gene, GADPH. The following primer sequences were designed using software Primer 5.0.

TLR4: forward: 5CAGGTCGAATTGTATCGCCTT3′

reverse: 5CCTGTGAGGTCGTTGAGGTTAG3′

IκBα: forward: 5CGAGCATTCTATTGTGGTGATTC3′

reverse: 5GCTACAGTCTATGGCGGTTCAA3′

IRAK4: forward: 5GACTTGCGGCTGTGGATG3′

reverse: 5TCTCGTGCAGACACTGGCTA3′

HuR: forward: 5CACAGTGAAGTTTGCAGCCA3′

reverse: 5TTGCCCAAGGTTGTAGATGAA3′

c-Myc: forward: 5′GCTCAAAGCCTAACCTCACAA3′

reverse: 5′AAAGAAAGAAGATGGGAAGCA3′

EMR1: forward: 5TCAAGGATACGAGGTTGCTGA3′

reverse: 5CTGAAGGCTGTTGATAGTGGTGA3′

GADPH: forward: 5′GGGAAACTGTGGCGTGAT3′

reverse: 5′GAGTGGGTGTCGCTGTTGA3′

#### Western blotting analysis

The common carotid was cut into slices, which were placed in an EP tube together with 100 µl of ice-cold RIPA lysis buffer supplemented with 1 µL 100 mM phenylmethanesulfonyl fluoride (PMSF; Beyotime Institute of Biotechnology; China) and dissociated on ice for 30 minutes after being homogenized 5 times. Protein samples were extracted and separated by SDS-polyacrylamide gel electrophoresis (SDS-PAGE), transferred onto polyvinylidene fluoride (PVDF) membranes, and blocked with TBST buffer. The membranes were incubated with anti-TLR4 (1∶3000), anti-IRAK4 (1∶1000), (cell signaling technology) anti-HuR (1∶2000) and anti-IκBα (1∶200) primary antibodies at 4°C overnight (Santa Cruz Biotechnology, USA) and blotted with horseradish peroxidase (HRP)-labeled secondary antibodies at 37°C for 2 h). Enhanced chemiluminescence (ECL) (Beyotime Institute of Biotechnology; China) developing methods were used. Image-Pro plus Version 6.0 for Windows (Media Cybernetics, Inc; USA) helped make measurements of band density. Three separate experimental results were obtained.

### Methods in BMM

#### Cell treatment

Bone marrows from tibia and femurs of Wistar and GK rats was washed with DMEM and cultured in DMEM with 20% heat-inactivated FBS and 30% L929 cell-conditioned medium for 5–7 days at 37°C in an air atmosphere containing 5% CO_2_ to promote the differentiation and proliferation. Cells were cultured in high glucose and normal nutrient media, respectively. LPS was used to stimulate BMM.

#### Western blotting analysis

Following LPS stimulation, cell proteins were extracted with PMSF and RIPA lysis buffer at 0, 15, 30 and 60 min and analyzed by western blotting analysis.

#### IL-6 by ELISA

Cells were stimulated with LPS and the supernatants were collected at 0, 2, 4, 12 and 24 h. IL-6 concentration in the culture media were measured by ELISA.

### Statistical analysis

GraphPad Prism 5.0 (GraphPad Software Inc.; California, USA) was used, all data being presented as mean ± SEM. Two-way analysis of variance (two-way ANOVA) was used for comparisons. p<0.05 was considered statistically significant.

## Results

### Blood glucose and lipid levels

The levels of GLU and CHOL in uninjured, Day 3 and Day 7 GK rats were higher than in Wistar rats, whereas TG was not statistical different. Blood glucose, total cholesterol and triglycerides levels were no statistical differences among the 3 groups of Wistar and GK rats in a response to catheter balloon injury (Table 1).

**Table 1:** Levels of serum GLU, CHOL and TG in GK rats and Wistar rats.

Groups Indexes	Wistar uninjured	GK uninjured	p-value	Wistar Day3	GK Day3	p-value	Wistar Day7	GK Day7	p-value
**GLU (mmol/L)**	12.01±0.37	19.39±1.05	p<0.01	13.43±0.53	19.22±1.07	p<0.01	13.47±0.50	18.23±0.29	p<0.01
**CHOL(mmol/L)**	1.20±0.10	2.37±0.61	p<0.01	1.43±0.14	2.55±0.16	p<0.01	1.21±0.22	3.00±0.14	p<0.01
**T G(mmol/L)**	0.62±0.08	1.79±0.59	p>0.05	0.94±0.24	1.23±0.32	p>0.05	0.89±0.33	0.85±0.20	p>0.05

GLU: blood glucose, CHOL: total cholesterol, TG: triglycerides.

### EVG staining

**Figure 2:**
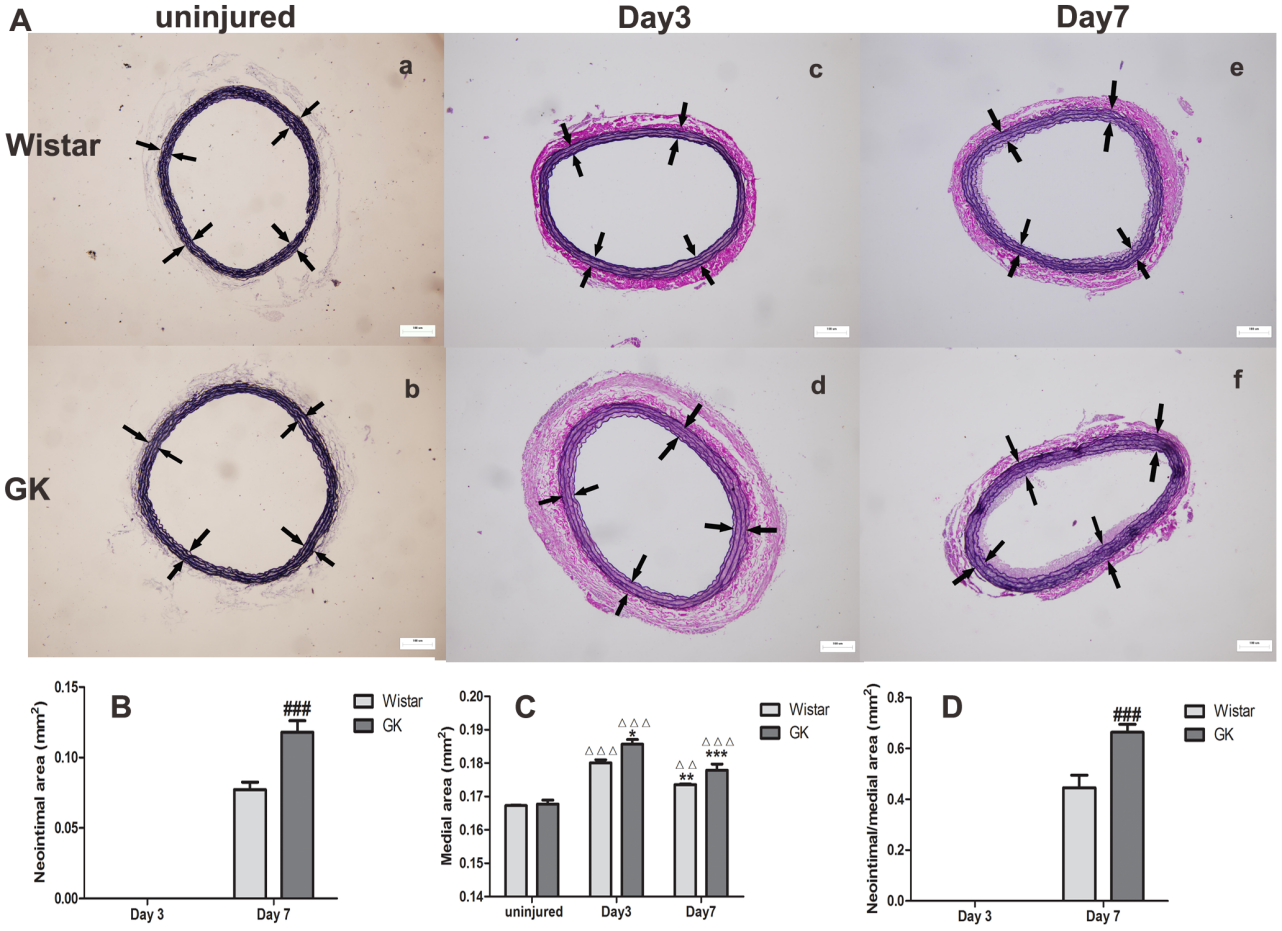
EVG staining of common carotid arteries in Wistar and GK rats (100×). EVG staining of uninjured (A-a, A-b), Day 3 post-injury (A-c, A-d) and Day 7 post-injury (A–e, A–f) in Wistar and GK rats. Neointimal emerged at Day 7 post-injury (B), the media peaked at Day 3 post-injury (C) and N/M ratio in GK rats was higher than that in Wistar rats (D) at Day 7 post-injury. White arrows pointed to the media. Data are represented as mean ± SEM. ^ΔΔΔ^indicates versus uninjured, p<0.001; *indicates versus Day 3 post-injury, p<0.05, **p<0.01; ^#^indicates versus Day 7 post-injury, p<0.05, ^###^p<0.001.

Intimal and medial changes were more easily monitored by EVG staining (Figure 2-A), especially with regards to the latter. Neointima appeared at Day 7 post-injury in both sets of rats (Figure 2-A–e, f, B). The medial area in Wistar rats peaked at Day 3 and decreased by Day 7 (p<0.001; p<0.01), with the same occurring in GK rats (p<0.05). The medial area at Day 3 and Day 7 in GK rats was greater than in Wistar rats, (p<0.01; p>0.05, respectively; Figure 2–C). The N/M at Day 7 in GK rats was significantly higher than in Wistar rats (p<0.001; Figure 2–D). HE staining is shown in supplementary files (Figure S1).

### EdU staining

**Figure 3:**
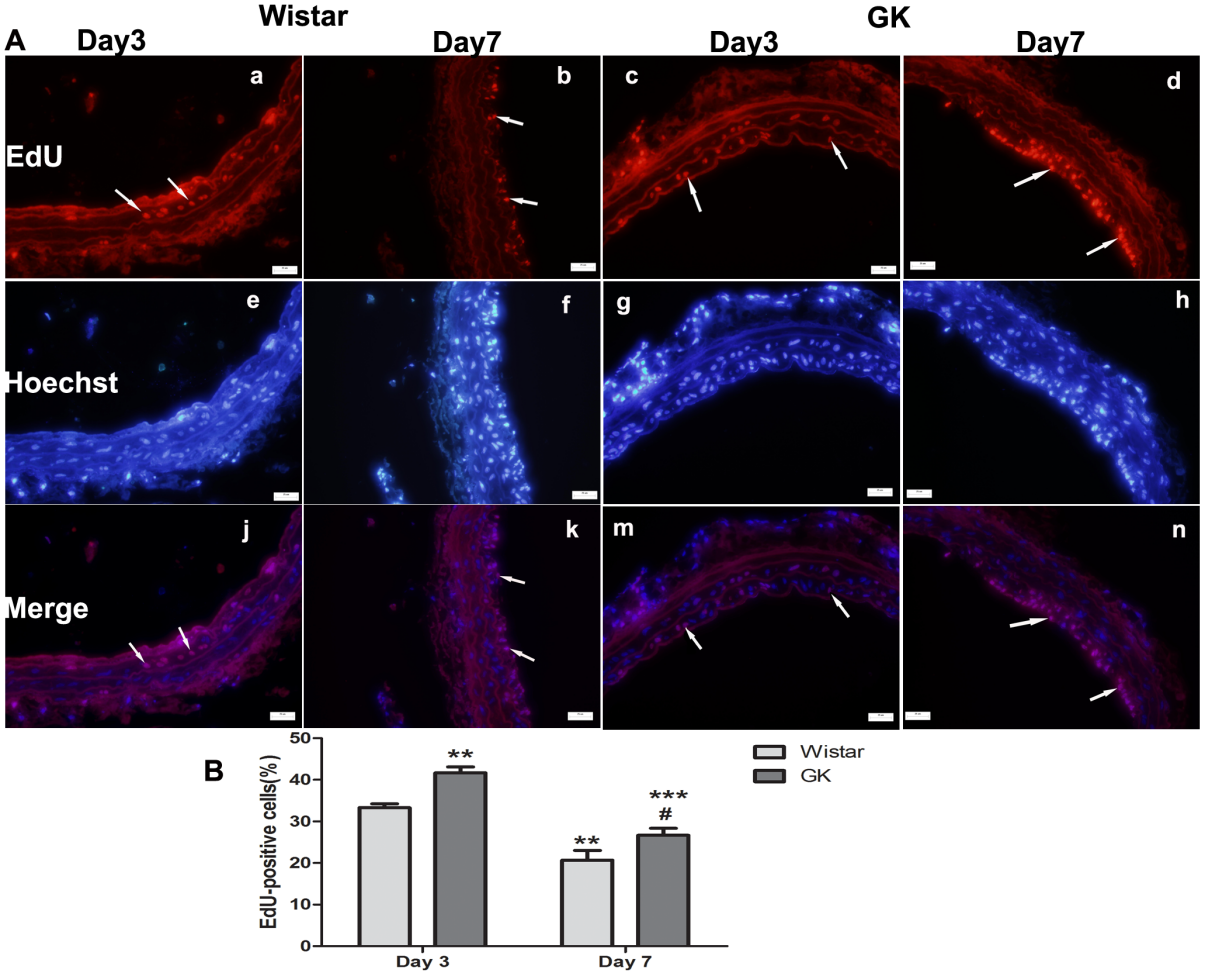
Representative images of EdU staining of common carotid arteries in Wistar and GK rats (400×). EdU staining (A–a, b, c, d), Hoechst33342 staining (A–e, f, g, h), images of merging EdU staining and Hoechst33342 staining (A–j, k, m, n). Percentage of EdU-positive cells of Day 3 post-injury were more than those of Day 7 post-injury in both rats and percentage of EdU-positive cells in GK rats were more than those in Wistar rats (B). Data are represented as mean ± SEM. **indicates versus Day 3 post-injury, p<0.01, ***p<0.001; ^#^indicates versus Day 7 post-injury, p<0.05.

The percent of EdU-positive cells (proliferating cells) seen at Day 3 in GK rats (Figure 3-A–c, g, m) was greater than in Wistar rats (Figure 3-A–a, e, j) and at Day 7 in GK rats (Figure 3-A–d, h, n; p<0.001). The percent of EdU-positive cells at Day 7 in Wistar rats (Figure 3-A–b, f, k) was lower than in GK rats (p<0.05; Figure 3–B).

### Immunohistochemistry for PCNA and NFκBp65

**Figure 4:**
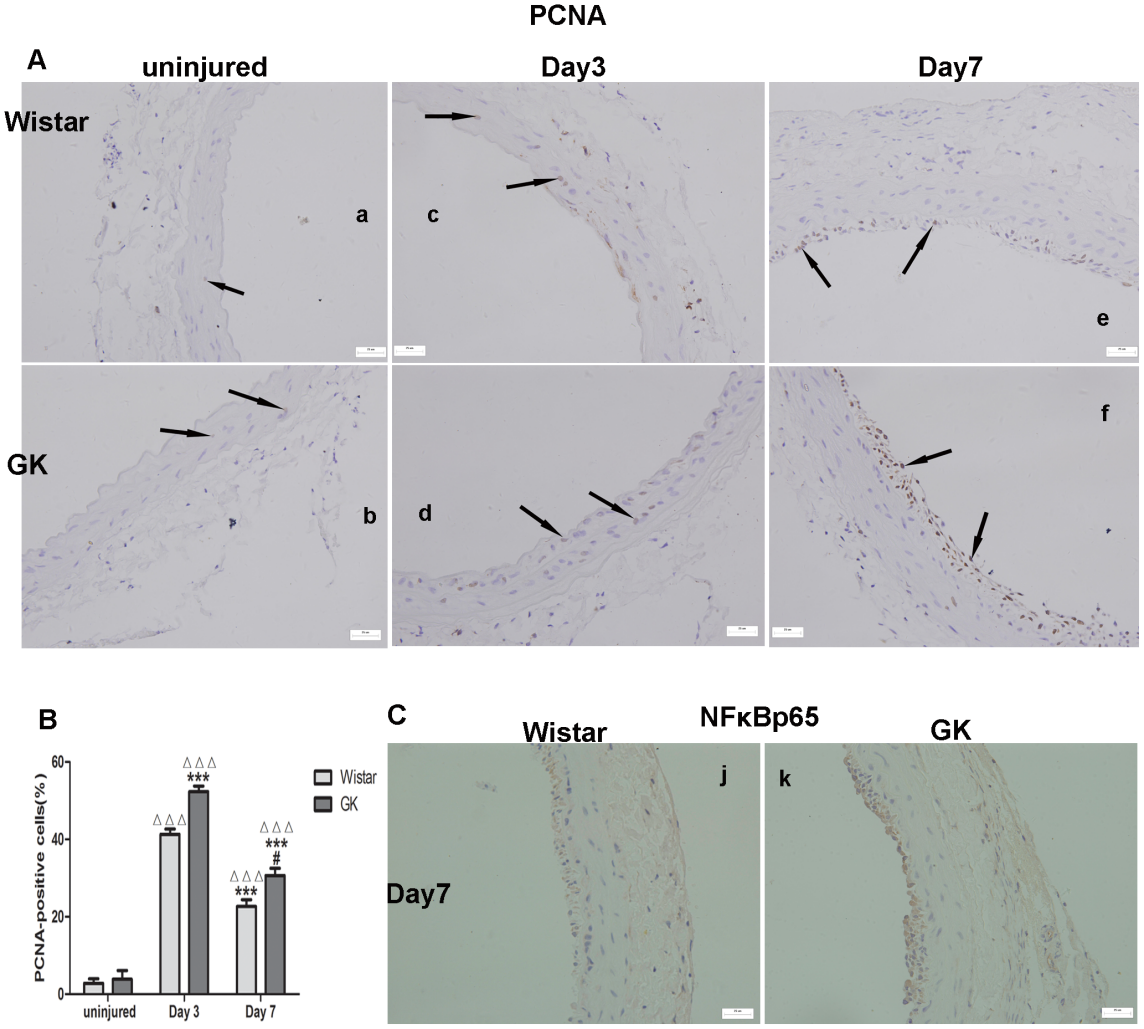
Immunohistochemistry for the expression of PCNA and NFκBp65 in common carotid arteries. PCNA-positive cells of uninjured in both rats (A–a, A–b). PCNA-positive cells of Day 3 post-injury (A–c, A–d) were more than those of Day 7 post-injury (A–e, A–f) in both rats and PCNA-positive cells in GK rats were more than those in Wistar rats (B). NFκBp65-positive cells (C) in GK rats were more than those in Wistar rats at Day 7 post-injury. Data are represented as mean ± SEM. ^ΔΔΔ^indicates versus uninjured, p<0.001; ***indicates versus Day 3 post-injury, p<0.001; ^##^indicates versus Day 7 post-injury, p<0.01.

There was no difference in the PCNA-positive cells between uninjured Wistar rats (Figure 4-A–a) and GK rats (Figure 4-A–b; p>0.05). More PCNA-positive cells at Day 3 and Day 7 post-injury were found in GK rats (Figure 4-A–d, f) than their Wistar counterparts (Figure 4-A–c, e; p<0.001; p<0.05, respectively). PCNA-positive cells were more numerous at Day 3 post-injury in both rats than at Day 7 post-injury (p<0.001; p<0.001; Figure 4–B). GK rats had more NFκBp65-positive cells than Wistar Day 7 post-injury rats (Figure 4–C). The positive and negative controls of PCNA and NFκBp65 have been presented (Figure S2).

### Agilent Whole Genome Oligo Microarray

**Figure 5:**
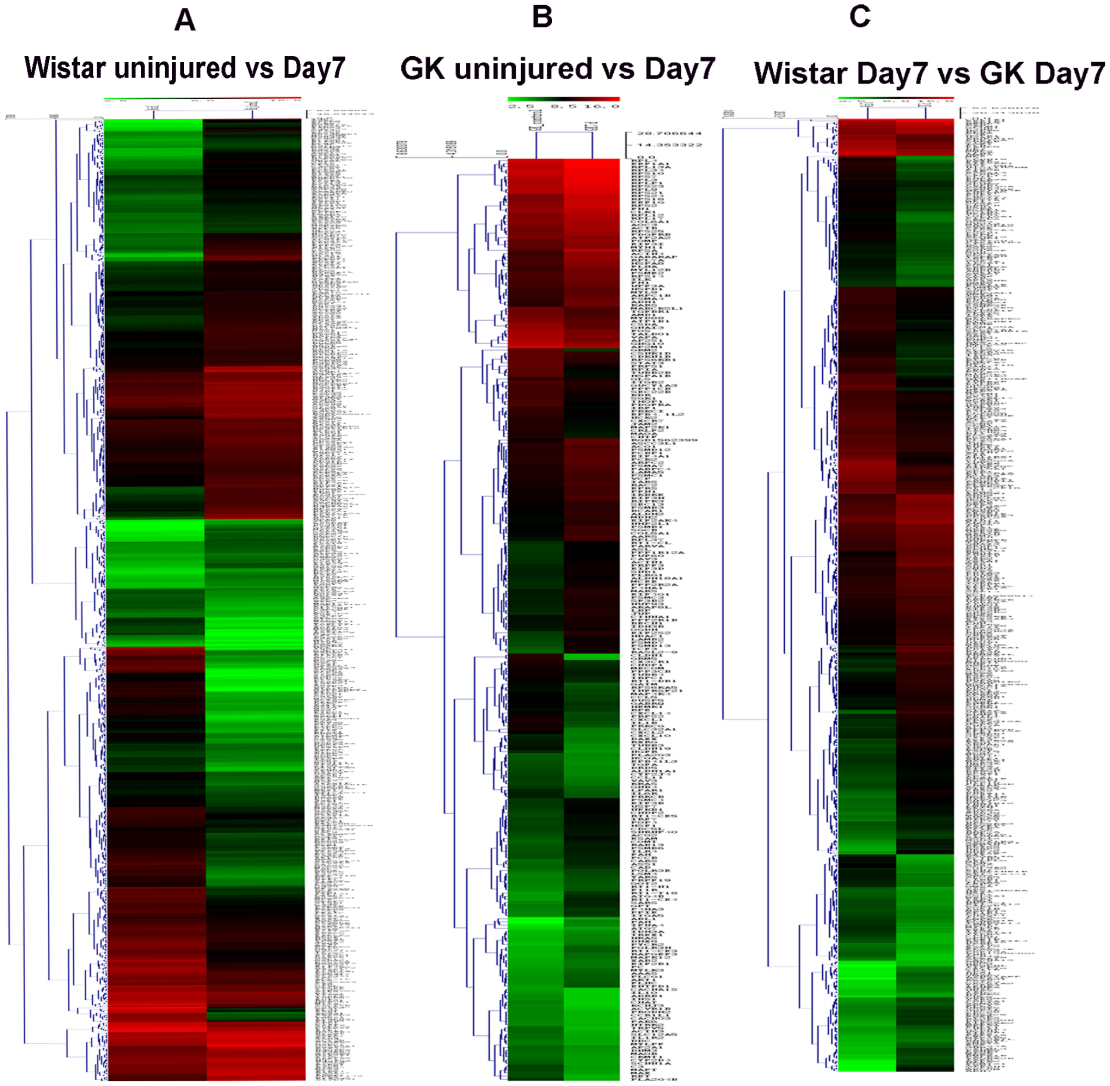
Agilent Whole Genome Oligo Microarray of common carotid arteries in Wistar and GK rats. Through three pairs of comparison (Wistar uninjured versus Wistar Day 7 post-injury (A), GK uninjured versus Day 7 post-injury (B), Wistar Day 7 post-injury versus GK Day 7 post-injury(C)), IL-1/TLR-induced NFκB activation signaling pathway was filtered out.

Gene microarrays (Figure 5) were used to identify genes whose expression was altered ≥2-fold in 3 pairs of comparisons (Wistar uninjured versus Day 7 (Figure 5–A), GK uninjured versus Day 7 (Figure 5–B), Wistar Day 7 versus GK Day 7 (Figure 5–C)). Microarray analysis showed that carotid injury leads to the activation of many signaling pathways, including the IL-1/TLR-induced NFκB signaling pathway.

### qRT-PCR

**Figure 6:**
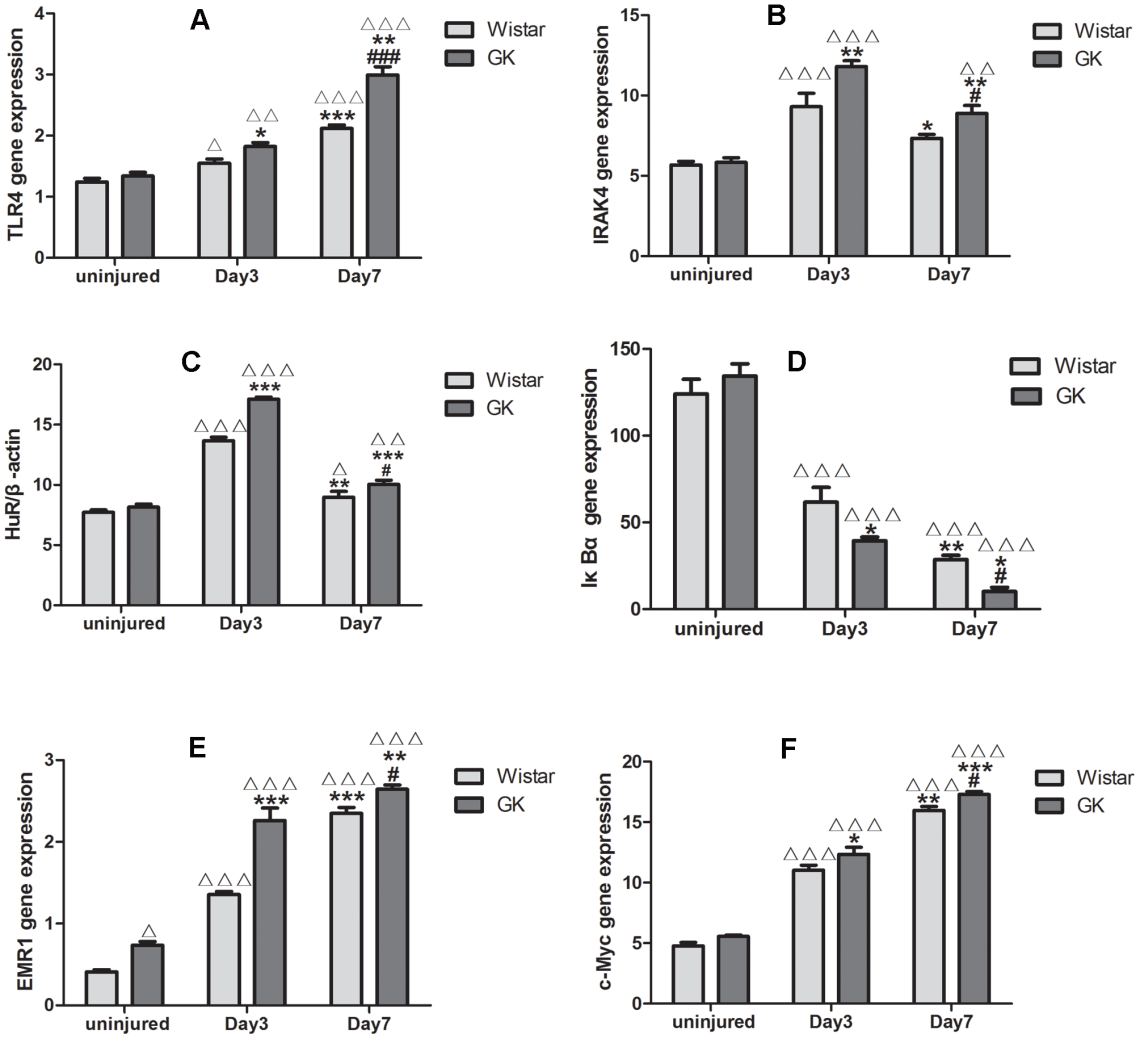
qRT-PCR of factors from common carotid arteries in Wistar and GK rats. mRNA levels of TLR4, IRAK4, HuR, IκBα, EMR1 and c-Myc in Wistar rats and GK rats(A, B, C, D, E, F). Data are represented as mean ± SEM. ^Δ^indicates versus uninjured p<0.05, ^ΔΔ^p<0.01, ^ΔΔΔ^p<0.001; *indicates versus Day 3 post-injury, p<0.05, **p<0.01, ***p<0.001; #indicates versus Day 7 post-injury, p<0.05, ^###^p<0.001.

qRT-PCR was used to detect the mRNA levels of some factors involved in the inflammatory response, and were also included in the IL-1/TLR-induced NFκB activation signaling pathway, such as TLR4, IRAK4, EMR1, IκBα, HuR, c-Myc. TLR4, EMR1 and c-Myc increased from the onset (Figure 6-A, E, F). IRAK4 and HuR peaked at Day 3 post-injury and then decreased (Figure 6-B, C). IκBα mRNA levels declined after injury (Figure 6-D). These factors are found in the injured carotid artery; their trends in the different groups were also consistent with our above-mentioned results.

### Western blotting analysis in vivo

**Figure 7:**
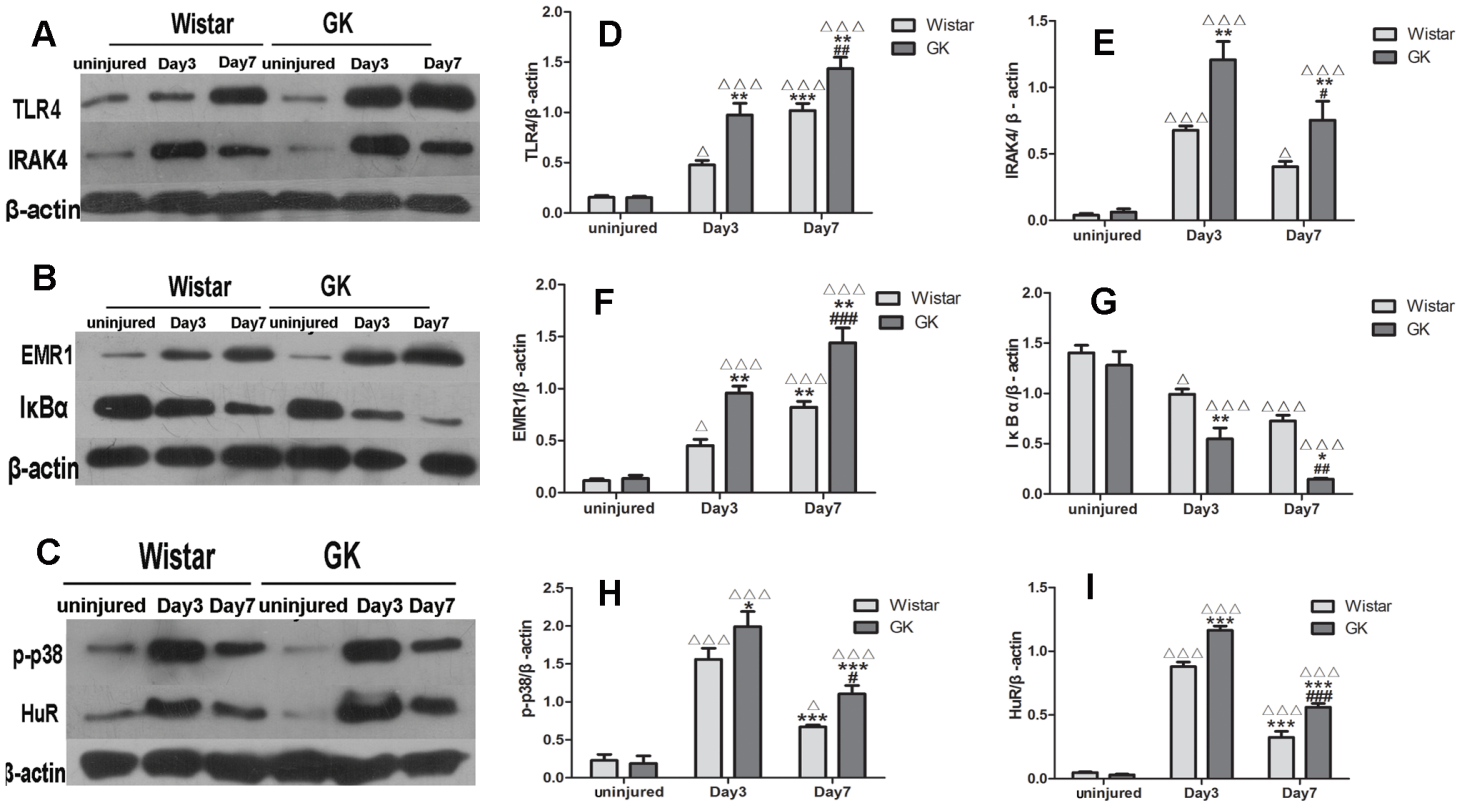
Changes of proteins included in IL-1/TLR-induced NFκB signaling pathway by western blotting analysis invivo. Representative western blotting of TLR4 and IRAK4 (A), EMR1 and IκBα (B), p-p38 and HuR(C). Bar diagrams depicting the relative protein level of TLR4, IRAK4, EMR1, IκBα, p-p38 and HuR (D, E, F, G, H, I). Data are represented as mean ± SEM. ^ΔΔΔ^indicates versus uninjured, p<0.001; *indicates versus Day 3 post-injury, p<0.05, **p<0.01, ***p<0.001;^ #^indicates versus Day 7 post-injury, p<0.05, ^##^p<0.01, ^###^p<0.001.

The levels of several proteins in the arteries of both sets of rats, including TLR4, IRAK4, IκBα, HuR, p-p38 and EMR1, were measured by Western blotting. Induction of TLR4 was a long-lasting growing process after injury, with levels peaking at Day 7 post-injury in both sets of rats, but with higher levels seen in GK rats than in Wistar rats (p<0.01; Figure 7-A, D). Similar results were seen with the EMR1 levels (p<0.001; Figure 7-B, F). However, IκBα protein levels decreased after the injury, with the rate of decline being more drastic in GK rats than in Wistar rats (Figure 7-B, G). With regards to the levels of IRAK4, p-p38 and HuR proteins, these peaked at Day 3 post-injury, with all 3 proteins being more abundant in GK rats than in Wistar rats (p<0.01; p<0.05; p<0.001, respectively; Figure 7-A, C, E, H, I).

### Western blotting analysis in BMM

**Figure 8:**
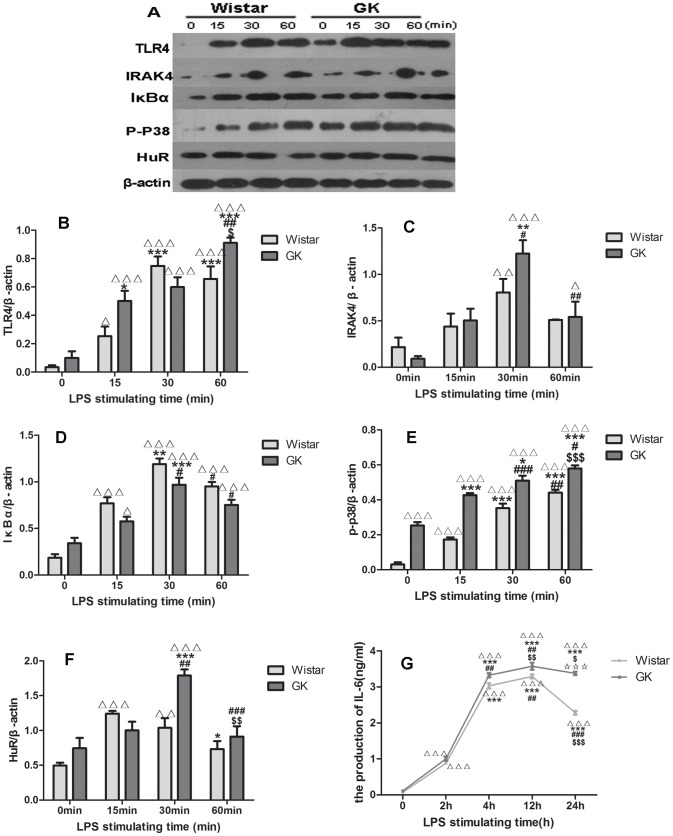
LPS-induced activation of factors in BMM. Representative western blotting bands of TLR4, IRAK4, IκBα, p-p38, HuR in BMM from Wistar rats and GK rats (**A**). Bar diagrams depicting the relative protein levels after normalization to β-actin (**B,** **C, D, E, F**). Changes of IL-6 with the LPS stimulation by ELISA in both rats (**G**). Data are represented as mean ± SEM. ^Δ^indicates versus 0 h, p<0.05, ^ΔΔΔ^p<0.001; *indicates versus 2 h, p<0.05, **p<0.01, ***p<0.001; ^#^ indicates versus 4 h, p<0.05,^ ##^p<0.01, ^###^p<0.001; ^$^indicates 12 h, p<0.05, ^$$^p<0.01,^ $$$^ p<0.001; ^☆^indicates 24 h, p<0.05, ^☆☆☆^p<0.001.

The levels of different proteins in BMM, including TLR4, IRAK4, IκBα, HuR and p-p38, were measured by Western blotting (Figure 8-A). After stimulation with LPS, the amounts of these proteins changed with time; compared with the maximum expression seen at 30 min in Wistar rats, TLR4 peaked at 60 min in GK rats with greater abundance (p<0.05; Figure 8-B). The levels of IRAK4, IκBα, p-p38 and HuR in GK rats were also higher than in Wistar rats (p<0.05; p<0.05; p<0.001; p<0.01, respectively; Figure 8-C, D, E, F).

### IL-6 assayed with ELISA

Production of IL-6 kept increased and peaked in both rats at 12 h LPS stimulation in BMM. At 12 h, GK rats reached higher levels than in Wistar rats. (p<0.01; Figure 8-G).

Some data in the research have been presented in supplementary files (Table S1).

## Discussion and Conclusion

Type 2 DM is not only a disease of carbohydrate metabolism, but a vascular disease [14]. Not only are the risks of diabetes equivalent to that of CAD, it is also an independent and has the greatest potential risk factor for cardiovascular events [15]. Patients with DM have an enhanced cardiovascular risk [16]. Compared with non-DM, DM patients with CAD have twice the rate of cardiovascular death rate and an increased incidence of heart failure. They were characterized with the metabolic derangements, the concomitant risk factor, e.g. hypertension, central obesity, dyslipidemia and abnormal endothelial function with reduced coronary flow reserve [17].

While PCI is now an established treatment for CAD [17], its major limitation, restenosis, remains a vexing problem [18], [19]. Maybe the angiography characters of DM with CAD - which include diffuse, extensive involvement of smaller reference vessels, multivessel involvement, higher incidence of left main coronary artery disease, poorer collaterals, and lower ejection fraction - promote the formation of restenosis and result in the poorer outcomes after revascularization in diabetic than non-diabetic patients [20].

Restenosis after stent deployment is an overreaction of the wound-healing response after vascular injury, and is characterized by the following sequence of events: inflammation, granulation, extracellular matrix remodeling, and vascular smooth muscle cell (VSMC) proliferation and migration [21]. Drug-eluting stent (DES) technology designed to reduce restenosisis among the great success stories in cardiology [22]. However, current DES is not wholly suitable for all CAD patients, especially for those with DM who suffer greater risks of restenosis.

TLR4 plays a major role in mediating cellular inflammation and VSMC proliferation, which is involved in both atherogenesis and restenosis [23]. However, the identity of the pathway(s) activated by TLR4 and the underlying mechanisms are unclear. We hypothesize that the IL-1/TLR-induced NFκB signaling pathway - in which TLR4 has key roles - promotes the development of inflammation.

We showed here inflammation and proliferation occurring after catheter balloon injury of Wistar and GK rats involve the IL-1/TLR signaling pathway. Compared with the Wistar rats, GK rats had more VSMC proliferation and vascular inflammation. NFκBp65 at Day 7 post-injury artery seems to verify this hypothesis. Microarray showed the IL-1/TLR-induced NFκB signaling pathway was activated in Wistar and GK rats after carotid injury. To elucidate the pathways activated by injury and clarify the mechanism, several factors in the IL-1/TLR-induced NFκB signaling pathway were chosen for investigation.

TLR4, one of TLRs, expressed in almost all cells and is usually activated in response to tissue injury by ligands such as cellular fibronectin, heat shock protein 60 and endogenous peptides [24], [25]. After injury or stimulation, the endogenous ligands increase, and IL-1R/TLR recruits the adaptor molecule myeloid differentiation factor 88 (MyD88) to their TIR domain, transducing more signaling in the cells. Disruption of the TLR4 gene in mice confers protection from obesity-induced inflammation and insulin resistance [26], [27]. In our carotid balloon injury and LPS stimulation BMM, western blotting and qRT-PCR showed long-lasting expression of TLR4, which confirm the inflammatory process and are consistent with c-Myc, EMR1 and PCNA levels. Kim has demonstrated that IRAK4 kinase-inactive knock-in mice are completely resistant to LPS- and CpG-induced shock, and this trait is due to impaired TLR-mediated induction of proinflammatory cytokines and chemokines [28]. We found a high level of IRAK4 expression in injured arteries at Day 3 that decreased by Day 7, which is consistent with Hatao’s results, who found that prolonged stimulation of TLRs caused a decrease in IRAK4 protein accompanied by the appearance of a smaller molecular weight protein. Activation of NFκB may result in cleavage of IRAK4 by a protease [29].

After the IRAK4 is activated, NFκB is finally activated by IκBα phosphorylation and degradation by its upstream IκB kinase (IKK)-α/β phosphorylation and IKK-β activation [30], [31]. As expected, a more robust down-regulation of IκBα following balloon injury at Day 3 as well as Day 7 occurred in GK rats.

At the same time IκBα is activated, p38 is phosphorylated, which then migrates to the nucleus. Therefore, HuR is phosphorylatedand shifts to the cytoplasmic compartment [32]. This nuclear-cytoplasmic shuttling of HuR may promote VSMC proliferation, the inflammatory response and neointimal formation [33], like our results with western blotting and qRT-PCR.

Activation of IL-1/TLR-induced NFκB pathway finally results in chemokine and cytokine production, e.g. IL-1β, TNFα, IL-12IL-6 [34]. Production of IL-6 shown by ELISA in our study proves this outcome. Cytokines and growth factors subsequently induce proliferation and migration of vascular smooth muscle cells [35]–[37]. Media-to-intima migration, proliferation of VSMCs and the subsequent synthesis of an extracellular matrix are the most critical stages in the pathogenesis of neointima formation [21]. We speculated that there is an increase in EdU-positive cells at Day 3 post-injury, a decrease in EdU-positive cells in media, and emergence of EdU-positive cells in the intima at Day 7 post-injury. HE (Figure S1) and EVG staining indicate this proliferation. We drew the conclusion that it was after Day 3 that medial proliferative cells migrated into the intima, and the neointima had just started to be produced at Day 7. Expression of factors in the IL-1/TLR-induced NFκB signaling pathway and of proliferation factors (PCNA, c-Myc and EMR1) are always more obvious in GK rats than Wistar rats.

There is also a crosstalk between the TLR and PI3K/Akt signaling pathways [38]. Stimulation of TLR2 results in recruitment of active Rac1 and PI3K to the TLR2 cytosolic domain, which leads in turn to activation of the PI3K/Akt pathway [39], a pathway that is involved in DM. Moreover, high blood glucose levels can also accelerate the proliferation and immigration. c-Myc has been implicated in the loss and dysfunction of insulin-producing β-cells; it appears to be a powerful trigger for β-cell apoptosis as well as loss of differentiation in rodent islets invivo [40], [41]. All the above may contribute to GK rats responding more strongly to inflammation than Wistar rats.

In conclusion, the study provides evidence supporting the hypothesis that the IL-1/TLR-induced NFκB signaling pathway is an essential mediator closely connected with the progression of vascular neointimal formation after catheter balloon injury in both Wistar and GK rats. Greater proliferation and inflammation occurs in GK rats than Wistar rats, which implies in human terms that DM patients progress further than non-DM patients after similar arterial injury.

Since the signalling pathway involved in the inflammation caused by carotid injury has been elucidated, anti-inflammation therapy should be the next step besides implementation of lifestyle modification and long-term weight loss.

It has been have confirmed that anti-inflammatory treatment decreases restenosis [42]–[44]. Anti-monocyte chemoattractant protein-1 (MCP-1) gene therapy could reduce the development of neointimal hyperplasia [45]. Dexamethasone-eluting stent [46] and Paclitaxe-eluting stent could inhibit coronary restenosis. Sirolimus, zotarolimus and everolimus, all potent immunosuppressive agents, inhibit SMC proliferation [47]. However, these and anti-inflammation therapies mentioned above are not the key method; they may interrupt cell-cycle, inhibit the SMC proliferation or non-specifically prevent inflammation.

If a target gene blocks the signaling pathway, the inflammation will be arrested at early stage before the release of chemokines and cytokines, and before SMC proliferation and immigration. This treatment could completely inhibit inflammation and may have other roles.

Although the critical signalling pathway has been discovered that gives some indication as to future direction for treatment, some lingering questions need further investigation. It could be more convincing to knock out one or more factors in the signaling pathway to demonstrate that IL-1/TLR-induced NFκB signaling pathway is correlated with intima hyperplasia after artery balloon injury in GK and Wistar rats. Which factor is the key factor-IRAK4, c-Myc or some factor else? This will be a long- term tough task and that is a critical therapeutic strategy for the prevention of vascular remodeling in proliferative vascular diseases, to which we are committed for the future.

## Supporting Information

Figure S1 **HE staining of common carotid arteries in Wistar and GK rats (100×).** HE staining (A), neointimal area (B), medial area (C), ratio of N/M (D). The media thickened at Day 3 post-injury(c, d) and neointimal emerged at Day 7 post-injury(e, f) in both rats. Ratio of N/M in GK rats was higher than that in Wistar rats. Data are represented as mean ± SEM. ^ΔΔΔ^indicates versus uninjured, p<0.001; *indicates versus Day 3 post-injury, p<0.05, **p<0.01, ***p<0.001; ^#^indicates versus Day 7 post-injury, p<0.05, ^###^p<0.001.
(TIF)

Figure S2 **Positive and negative controls for histological stainings (400×).** PCNA negative controls of Day 7 post-injury in Wistar (A) and GK rats (B). PCNA-positive cells in liver cancer (C) controls as the positive control. NFκBp65 negative controls of Day 7 post-injury in Wistar (D) and GK rats (E). NFκBp65-positive cells from lung tissue in asthmatic rats (F) as the positive control.
(TIF)

Table S1 (XLS)
