# Supplementary figures and images for: Interleukin-1/Toll-Like Receptor-Induced Nuclear Factor Kappa B Signaling Participates in Intima Hyperplasia after Carotid Artery Balloon Injury in Goto-Kakizaki Rats: A Potential Target Therapy Pathway
Source: PLoS One. 2014 Aug 1;9(8):e103794. doi: 10.1371/journal.pone.0103794 (PMC4118962; doi:10.1371/journal.pone.0103794)

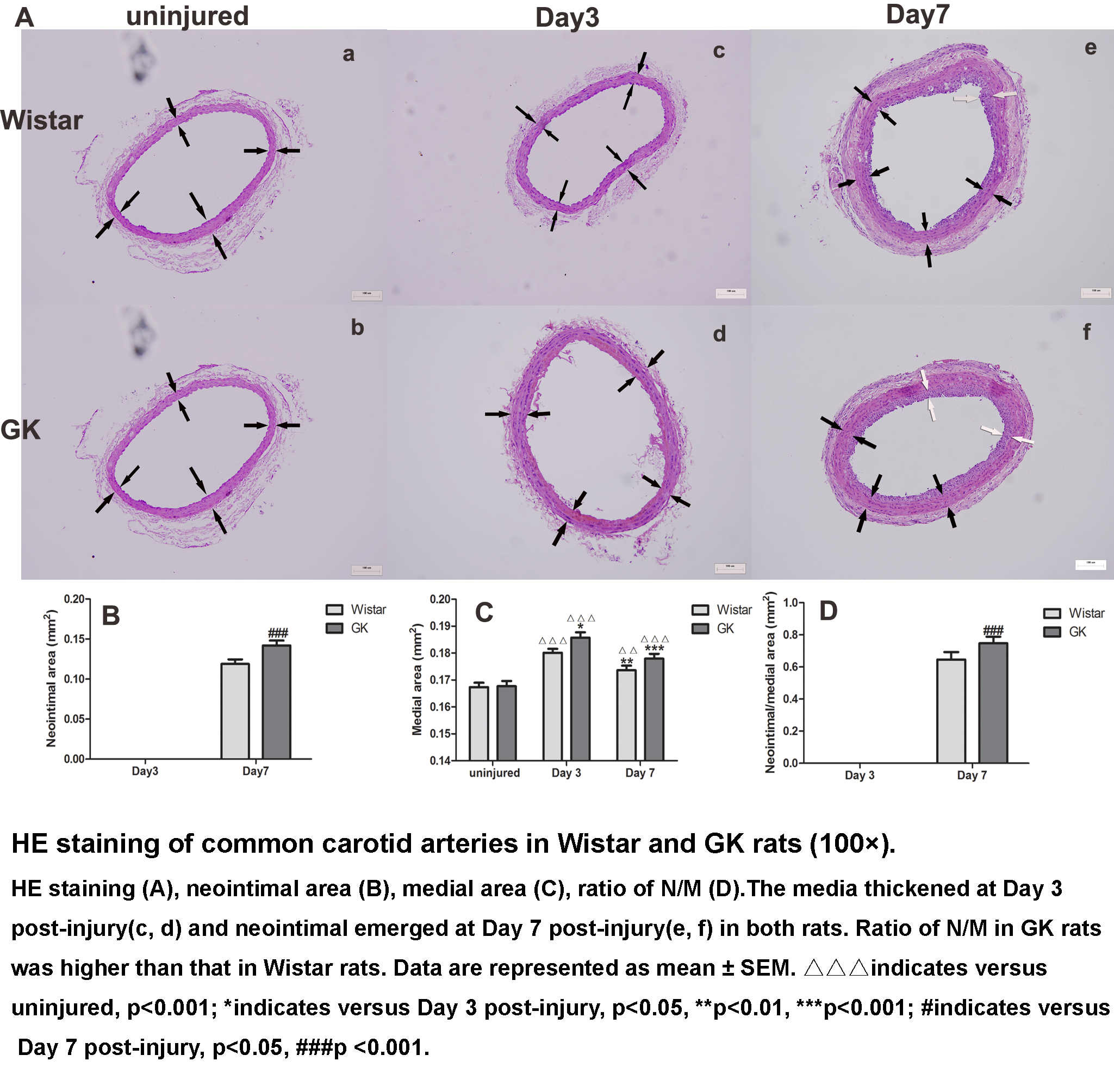

Supplement: Figure S1 — HE staining of common carotid arteries in Wistar and GK rats (100×). HE staining (A), neointimal area (B), medial area (C), ratio of N/M (D). The media thickened at Day 3 post-injury(c, d) and neointimal emerged at Day 7 post-injury(e, f) in both rats. Ratio of N/M in GK rats was higher than that in Wistar rats. Data are represented as mean ± SEM. ΔΔΔindicates versus uninjured, p<0.001; *indicates versus Day 3 post-injury, p<0.05, **p<0.01, ***p<0.001; #indicates versus Day 7 post-injury, p<0.05, ###p<0.001. (TIF) [file pone.0103794.s001.tif]

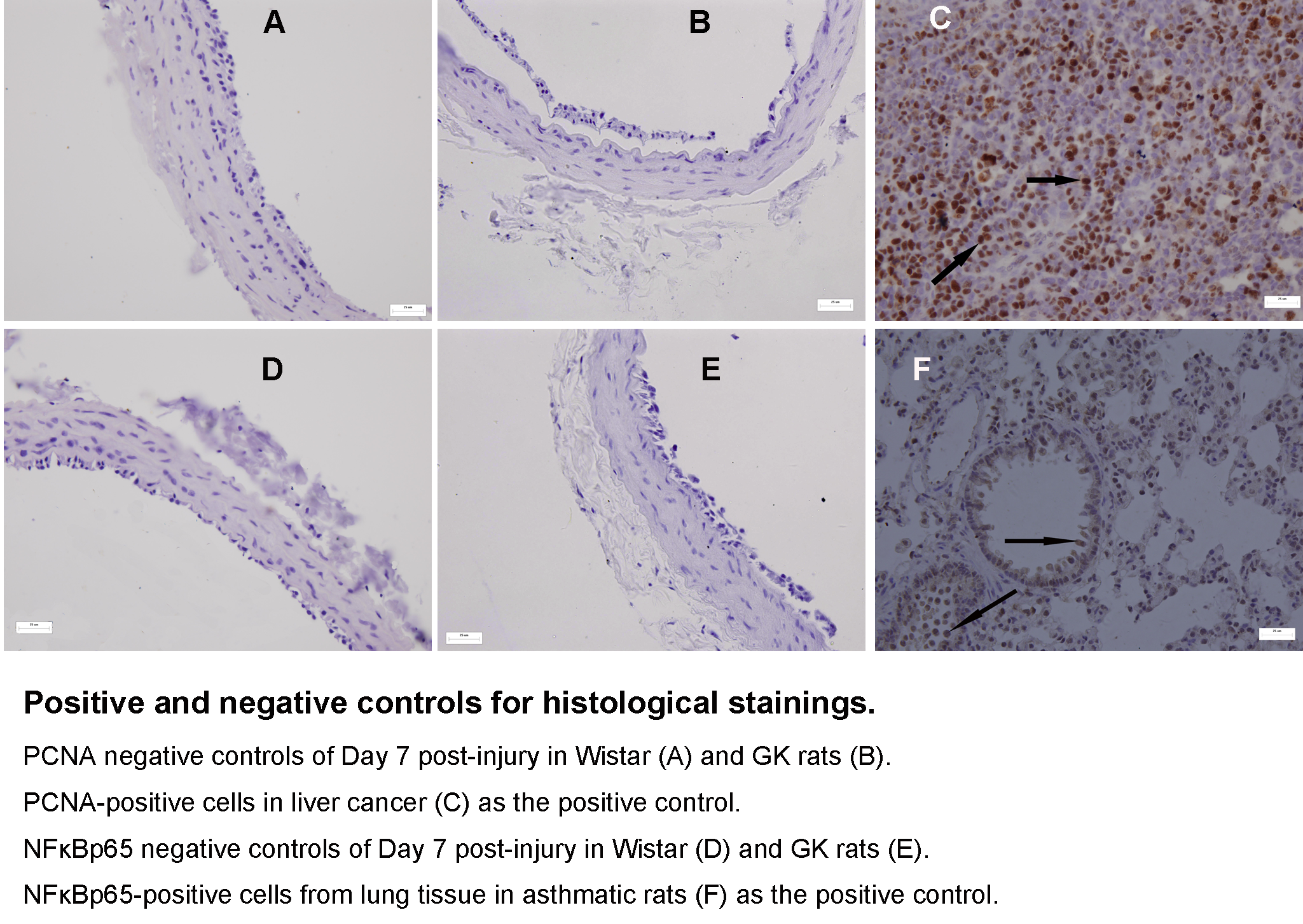

Supplement: Figure S2 — Positive and negative controls for histological stainings (400×). PCNA negative controls of Day 7 post-injury in Wistar (A) and GK rats (B). PCNA-positive cells in liver cancer (C) controls as the positive control. NFκBp65 negative controls of Day 7 post-injury in Wistar (D) and GK rats (E). NFκBp65-positive cells from lung tissue in asthmatic rats (F) as the positive control. (TIF) [file pone.0103794.s002.tif]
